# The Potential Role of Torsemide in Optimizing Loop Diuretic Therapy for Heart Failure Patients

**DOI:** 10.7759/cureus.41957

**Published:** 2023-07-16

**Authors:** V. K Chopra, P. P Mohanan, Vijay Kher, Raja Ram Mantri, Rajan Isaacs, Uday Jadhav, Nitin Zalte, Amarnath Sugumaran, Senthilnathan Mohanasundaram

**Affiliations:** 1 Clinical Cardiology Heart Failure and Research, Max Super Speciality Hospital, New Delhi, IND; 2 Cardiology, Westfort Hi-Tech Hospital, Thrissur, IND; 3 Nephrology, Medanta Kidney and Urology Institute, Gurugram, IND; 4 Cardiology, Sir Ganga Ram Hospital, New Delhi, IND; 5 Nephrology, Deep Kidney Care Centre, Ludhiana, IND; 6 Cardiology, Mahatma Gandhi Mission (MGM) New Bombay Hospital, Navi Mumbai, IND; 7 Medical Affairs, Cipla Ltd., Mumbai, IND

**Keywords:** optimal management, expert opinion, torsemide, loop diuretics, heart failure

## Abstract

Heart failure is associated with an increased frequency of hospitalization, reduced life span, and greater risk to public health, thus posing a challenge. In India, torsemide is one of the commonly used loop diuretics for decongestion in heart failure. However, this use of torsemide, including its dosing, and up/down titration, is based on practical experience. Loop diuretic therapy for heart failure patients poses several dilemmas due to the lack of robust evidence based on which treatment decisions can be made. To guide physicians on the optimal use of torsemide in heart failure patients with or without renal impairment, a panel of expert cardiologists and nephrologists from India convened to develop this expert opinion document for the use of torsemide. This expert opinion on torsemide will pave the way for optimal management with loop diuretic therapy in real-world heart failure patients.

## Introduction and background

Chronic heart failure (HF) is a progressive and debilitating complex clinical syndrome that affects millions of people worldwide. There is scarce epidemiological data from India regarding the burden of HF, however, a study by Huffman et al. has estimated a range of 1.3 to 4.6 million HF patients in India [1]. A total of 1.8 million annual hospitalizations in India are due to HF [2].

HF is a serious condition resulting from structural or functional abnormality of the heart in which the heart is unable to perform the required circulatory function [3-5]. HF is associated with an increased re-hospitalization, affects the quality of life and survival, and poses a challenge to public health with a substantial burden on healthcare systems and resources [3].

Acute HF (AHF) is a sudden or gradual onset of new or worsening signs and symptoms of HF. It is usually a serious condition that requires urgent medical attention. Patients with AHF need prompt evaluation and treatment that majorly focus on managing fluid overload and hemodynamic compromise [4-6]. Major clinical presentations in patients with AHF include acute decompensated HF, acute pulmonary edema, isolated right ventricular failure, and cardiogenic shock. All these clinical presentations are based on the presence of signs of congestion and/or peripheral hypoperfusion [5]. The majority of AHF patients develop peripheral and pulmonary congestion over days or weeks before hospitalization [7]. Congestion resulting in HF decompensation is a predictor of poor patient outcomes. To help patients achieve optimal volume status, the European Society of Cardiology (ESC) guidelines recommend treatment of the signs and symptoms of congestion. However, 50% of patients admitted for AHF have residual congestion at discharge resulting in re-hospitalization (up to 25% are re-hospitalized within a month of discharge) and death within a span of six months after discharge, regardless of the underlying pathology [7,8].

Major guidelines recommend the use of loop diuretics to alleviate signs and/or symptoms of congestion in HF patients. Loop diuretics have dual benefits, namely, to maintain euvolemia for chronic HF patients and to achieve decongestion in acute decompensated HF (ADHF) patients. Intravenous (IV) loop diuretics are administered in hospitalized patients, while sequential therapy with oral loop diuretics is followed and prescribed at discharge [4,5].

Among the loop diuretics, torsemide has distinct pharmacological properties, including predictable oral bioavailability (80%-100%), a pre-dominant hepatic metabolism, absorption that is not affected by food, a relatively long half-life (3-6 hours), and a quick onset of action. Additionally, torsemide can slow or reverse the development of myocardial fibrosis and attenuate progressive ventricular dilation and hypertrophy [9-13]. While loop diuretics can help manage symptoms of fluid overload, their role in preventing or treating cardiac remodeling in HF is not very clear. Studies have reported that loop diuretics can potentially reverse myocardial fibrosis and reduce collagen type I synthesis, particularly procollagen type I carboxy-terminal proteinase (PCP)) in patients with chronic HF [10,11].

In India, torsemide is one of the commonly used loop diuretics for decongestion in HF. However, the use of torsemide, including its dosing, up-titration, and down-titration is based on practical experience. The loop diuretic therapy poses several dilemmas due to the lack of robust evidence based on which treatment decisions can be made. Thus, obtaining an expert opinion on the use of torsemide, both orally and intravenously, can help optimize loop diuretic therapy for HF patients in real-world settings. Therefore, a panel of expert cardiologists and nephrologists from India convened to develop this expert opinion document to guide physicians on the optimal use of torsemide in HF patients, with or without renal impairment.

## Review

Oral torsemide and HF

Guidelines from major cardiology associations recommend oral loop diuretics such as torsemide to alleviate congestion, improve signs and/or symptoms, prevent HF worsening, improve exercise capacity, and reduce the risk of HF hospitalizations (Class I) [4,5].

Clinical evidence

Sheen et al. reported a significantly lower total urine volume (from 12 to 24 hrs. of diuretic administration) after furosemide 40mg compared with placebo or torsemide 20 mg [14]. In 2002, an open-label post-marketing surveillance study (TORIC Study) in HF patients demonstrated a lower risk of mortality and improvement in NYHA functional class in the torsemide group compared to the furosemide group. In addition, the study reported a lower incidence of hypokalemia in HF patients receiving torsemide compared to furosemide [15].

Furthermore, in another similar study in HF patients, torsemide exhibited greater efficacy in improving NYHA functional class and improved quality of life compared with furosemide [16]. The TORNADO Study showed that 94% of HF patients on torsemide reached a composite of improvement in NYHA Class, improvement of at least 50 m on 6 MWT, and a decrease of at least 0.5 ohms in fluid retention compared to 58% of patients on furosemide [17].

A network meta-analysis (READY) showed a significant reduction (60%) of HF-related hospitalization with torsemide treatment compared with furosemide. Whilst no significant difference in all-cause mortality, CV mortality, and the incidence of hypokalemia was exhibited between the loop diuretics [18].

Furthermore, a recent trial by Mentz et al. (TRANSFORM-HF) reported comparable effectiveness of mortality and hospitalization among torsemide and furosemide in HF patients. Torsemide reported a 6% reduction in total hospitalizations and an 8% reduction in all-cause mortality or hospitalization compared with furosemide [19].

Torsemide: benefits beyond diuresis and decongestion

A study by Harada et al. reported a significant reduction in ventricular wall stress associated with plasma aldosterone level with torsemide [20,21]. Another study by Lo´pez et al. showed a reduction in myocardial collagen accumulation and collagen type I synthesis in torsemide-treated patients [10].

Oral torsemide in ADHF post-discharge

Mentz et al. evaluated the use of loop diuretics (torsemide and furosemide) in AHF post-discharge and its association with baseline and post-discharge outcomes using the ASCEND study data. Patients treated with torsemide showed features of more severe disease. A strong association between torsemide use and elevated blood urea nitrogen, lower systolic blood pressure, and jugular venous distension was observed in the study. This suggests the trend of using torsemide in the scenarios of refractory volume overload and renal dysfunction by clinicians. A lower 30-day mortality/HF hospitalization and 180-day mortality were observed in the group treated with torsemide compared to furosemide [22].

**Figure 1:**
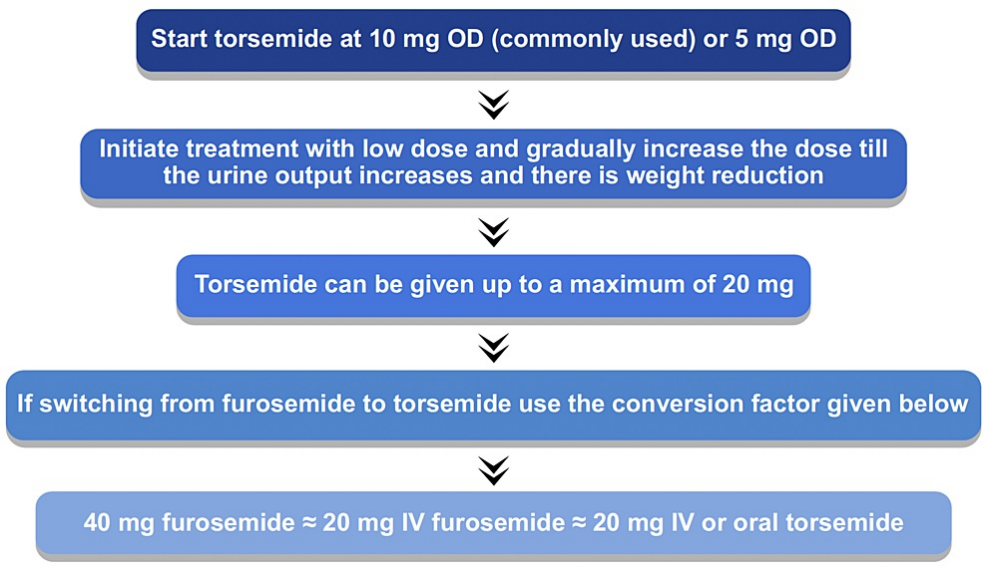
Oral torsemide dosing algorithm

**Figure 2:**
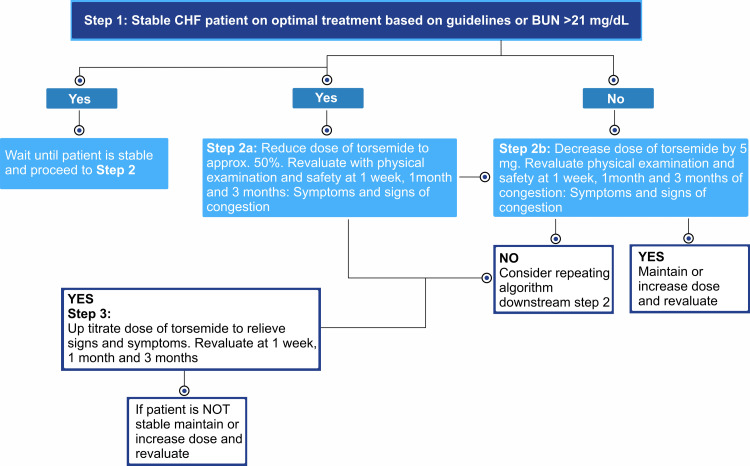
Dosing algorithms of torsemide in patients with heart failure

**Figure 3:**
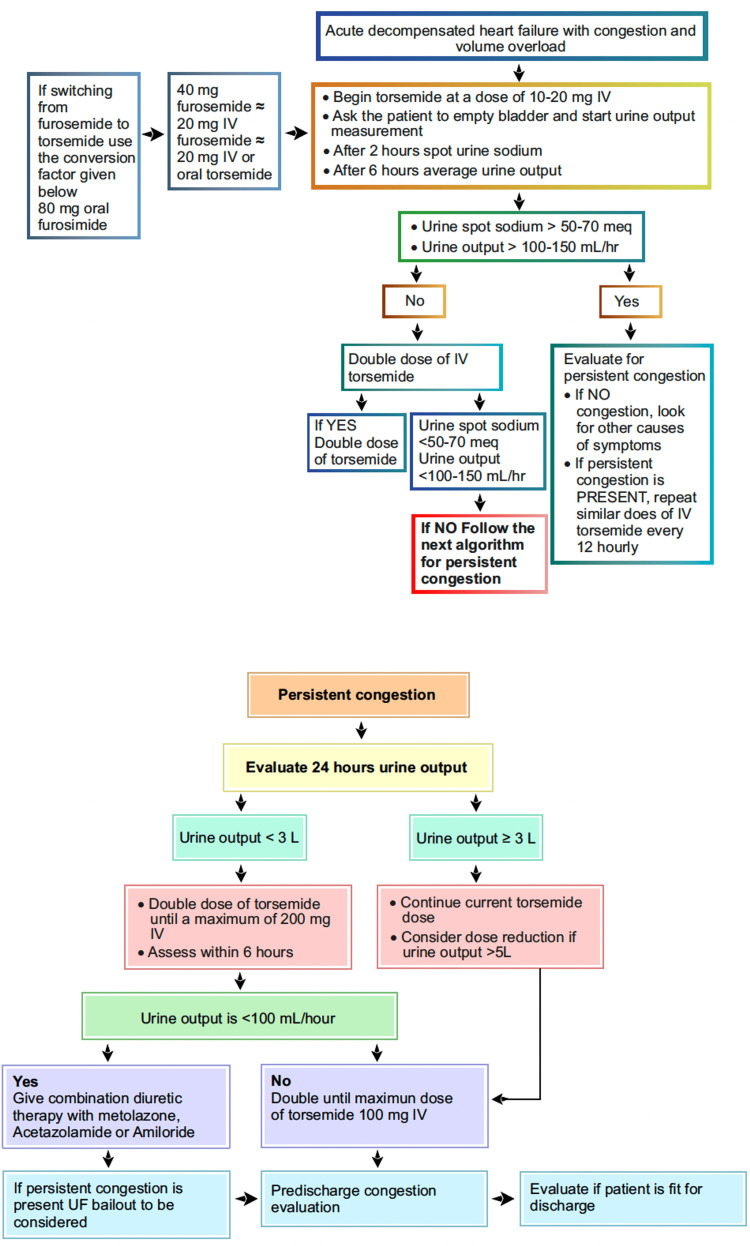
Parenteral torsemide-dosing algorithm

In an open-label study on HF patients, treatment with torsemide reported significantly fewer duration of HF hospitalization and HF rehospitalization compared to furosemide [23]. The dosing algorithms of torsemide in HF are listed in Figures 1-3.

IV loop diuretics

Parenteral loop diuretics are the mainstay of therapy in patients hospitalized with ADHF with signs of fluid overload and congestion. Clinical evidence has demonstrated that IV administration of loop diuretics results in the alleviation of symptoms and prompt diuresis in ADHF patients [24]. However, the ideal IV diuretic dose in ADHF patients is yet to be established. Treatment with above the guideline-recommended IV dose reported a higher 60-day mortality rate, whilst lower than guideline-recommended IV diuretic dose was associated with a longer hospital stay [25].

A study by Hariman et al. randomized 49 HF patients to receive a single IV dose of 5, 10, or 20 mg torsemide or 40 mg furosemide. A dose-related reduction in body weight and increase in sodium and chloride excretion and urine volume was produced by torsemide. A significant reduction in body weight was reported with 20 mg IV torsemide and 40 mg dose of furosemide. In addition, a significant increase in total and fractional 24-hour urinary excretion of sodium, chloride, and potassium and urine volume was reported with torsemide and frusemide doses [26]. Short courses of IV diuretics for volume management in patients with HF have been observed to be safe. They produce significant urine output and weight loss across different diuretic doses and ejection fractions.

Expert opinion recommendations

Oral torsemide should be preferred to furosemide in patients with HF due to its better bioavailability, longer half-life, higher potency, more robust and predictable natriuresis, and diuretic effect, particularly in comparison with the variable bioavailability of furosemide. IV furosemide and torsemide at equivalent doses are equipotent in the degree of natriuresis and diuresis. Higher torsemide doses will be required in patients with associated kidney failure, nephrotic syndrome, and hypoalbuminemia. Oral torsemide may be a favorable alternative in patients who are unresponsive in terms of signs and symptoms of congestion, body weight reduction, improvement in NYHA functional class, and 6 MWT. Oral torsemide may also be considered in patients at high risk of hospitalization for HF. Torsemide may reduce LV systolic wall stress without activating the sympathetic nervous system in asymptomatic or mildly symptomatic patients with chronic HF. This effect may be related to its anti-aldosterone properties. Torsemide may result in a reduction of myocardial collagen accumulation and a diminution of collagen type I synthesis in patients with HF, which may help halt or prevent cardiac fibrosis and myocardial structure changes, which are the end-stage pathologies observed in HF patients. Oral torsemide may improve LV diastolic function and decrease the plasma B-type natriuretic peptide (BNP) concentration in patients with chronic HF already receiving angiotensin-converting enzyme inhibitor (ACEI). The mechanism of action may be related to a dose-dependent blockade of aldosterone receptors by torsemide. In ADHF patients post-discharge, torsemide may be considered among those patients who have more severe disease in terms of symptoms, history of HF hospitalization, and renal impairment. In ADHF patients post-discharge who are recommended torsemide, the following dose conversion chart should be used: 40 mg furosemide ≈ 20 mg IV furosemide ≈ 20 mg IV or oral torsemide. In HF patients who are hospitalized with congestion and require IV loop diuretic therapy, IV torsemide may be considered in those patients with more severe HF and who fail to respond to conventional IV loop diuretic therapy in terms of signs and symptoms of congestion, body weight reduction and improvement in NYHA class. IV torsemide in HF can be administered in bolus doses. Continuous infusion may be attempted if there is no response to the bolus dose or if diuretic resistance is suspected.

## Conclusions

The prudent use of loop diuretics in patients with HF will require consideration of the physiological effects, as well as the pharmacokinetic and pharmacodynamic properties of the available drugs. Oral torsemide dose differs from furosemide in terms of better bioavailability and more robust and predictable diuretic effect, particularly in comparison with the variable bioavailability of furosemide. It is conceivable that these consistent diuretic effects of torsemide would translate into clinical benefits of reducing the need for HF hospitalization and facilitating easier outpatient HF management.
